# Dysphagia in neurological diseases: a literature review

**DOI:** 10.1007/s10072-020-04495-2

**Published:** 2020-06-07

**Authors:** M. Panebianco, R. Marchese-Ragona, S. Masiero, D. A. Restivo

**Affiliations:** 1 Department of Molecular and Clinical Pharmacology, Institute of Translational Medicine, University of Liverpool, L69 3BX, Liverpool, UK; 2 ENT Department, University of Padova, Via 8 Febbraio 1848, 2, 35122 Padova, PD Italy; 3 School of Physical Medicine and Rehabilitation, University of Padova, Via 8 Febbraio 1848, 2, 35122 Padova, PD Italy; 4 Neurology Department, Garibaldi Hospital, Catania, Italy

**Keywords:** Dysphagia, Swallowing, Neurological diseases

## Abstract

Dysphagia is defined as an impairment of this complex and integrated sensorimotor system. It is estimated that 400,000 to 800,000 individuals worldwide develop neurogenic dysphagia per year. Neurogenic dysphagia is typically occurring in patients with neurological disease of different etiologies. A correct and early diagnosis and an appropriate management of dysphagia could be useful for improving patient’s quality of life and may help to prevent or delay death. In the present review, we discuss thoroughly the anatomy and physiology of swallowing and also the pathophysiological mechanisms involved in impaired swallowing, as well as the diagnosis, management, and potential treatments of neurogenic dysphagia. Assessment of neurogenic dysphagia includes medical history, physical exam, and instrumental examinations (fiberoptic endoscopic evaluation of swallowing, videofluoroscopic swallowing study, electromyography). Pharmacological treatment of these problems includes oral anticholinergic drugs. Surgical myotomy of the cricopharyngeal muscle showed an important improvement of oropharyngeal dysphagia associated to upper esophageal sphincter hyperactivity. Chemical myotomy of the upper esophageal sphincter by local injections of botulinum toxin type A into the cricopharyngeal muscle has been proposed as an alternative less invasive and less unsafe than surgical myotomy.

## Introduction

Swallowing is defined as the semiautomatic motor action of the muscles of respiratory, oropharyngeal, and gastrointestinal tract that propels the food from oral cavity to the stomach and protects airway from food, liquids, and other substances. During a swallow, different levels of the central nervous system from the cerebral cortex to the medulla oblongata are involved. About 50 pairs of striated cranial muscles are excited and/or inhibited sequentially allowing the bolus transit from the mouth to the stomach. Dysphagia is defined as an impairment of this complex and integrated sensorimotor system. Neurogenic dysphagia (ND) is typically occurring in patients with neurological disease of different etiologies (see Table 1), and it is associated to high mortality, morbidity, and social costs [1–16]. Neurological problems that cause dysphagia can be categorized in many different ways: anatomic location of the lesion (e.g., central nervous system, peripheral nervous system or muscle), pathogenetic mechanism of disease (e.g., ischemic injury or degenerative process), etiology, or clinic presentation (e.g., dementia or movement disorders). A correct and early diagnosis and an appropriate management of dysphagia could be useful for improving patient’s quality of life and may help to prevent or delay death. In the present review, we discuss thoroughly the anatomy and physiology of swallowing and also the pathophysiological mechanisms involved in impaired swallowing, as well as the diagnosis, management, and potential treatments of neurogenic dysphagia.

**Table 1:** Dysphagia in neurological diseases

Degenerative	
Multiple sclerosis (MS)	
Parkinson’s disease (PD)	
*Parkinsonisms* Progressive supranuclear palsy (PSP) Multiple system atrophy (MSA) Spinocerebellar ataxia (SCA)	
*Dementia* Alzheimer’s disease (AD) Corticobasal degeneration Frontotemporal dementia Lewy body dementia Vascular dementia	
Huntington’s disease	
Wilson’s disease	
*Motor neurone disease (MND)* Amyotrophic lateral sclerosis (ALS) Primary lateral sclerosis	
Muscular dystrophy	
*Myophaties* Nemaline myopathy Mitochondrial myopathy Inclusion body myositis Polymyositis	
*Peripheral neuropathies* Inflammatory systemic neuropathies Diabetic neuropathy	
Nondegenerative	
*Vascular* Hemorrhagic stroke Ischemic stroke	
*Traumatic* Head injury	
N*eoplastic* Brain tumor	
*Congenital* Cerebral palsy	
*Iatrogenic (medication induced)* Tardive dyskinesia and dystonia	

The names in italic are the groups of diseases, but it is easily deductible for the readers

### Epidemiology

It is estimated that 400,000 to 800,000 individuals worldwide develop neurogenic dysphagia per year [17]. The reported incidence of dysphagia in specific neurologic diseases is variable, owing in part to patient selection methods and evaluation methods (e.g., questionnaires, clinical evaluation, diagnostic evaluation). It is generally agreed that stroke is the most common cause of ND. It is estimated that dysphagia occurs in approximately 65% of acute stroke patients. In Parkinson’s disease (PD), dysphagia occurs approximately in 50%. Dysphagia in multiple sclerosis (MS) occurs in 31.3%. Dysphagia is common in dementia with prevalence rates varying from 13 to 57%. Dysphagia is reported to be prevalent in 30–100% of individuals depending on type of motor neuron disease (MND) and the stage of disease affecting all individuals in the later stages of the disease. There are no data for less common neurological conditions. Moreover, malnutrition and aspiration pneumonia are the most common and troublesome consequences of dysphagia, with increased risk of death in elderly and debilitated patients. Especially, aspiration pneumonia is the most common cause of mortality in patients with neurological disease associated to dysphagia [18].

### Anatomy and physiology of the deglutition

**Fig. 1:**
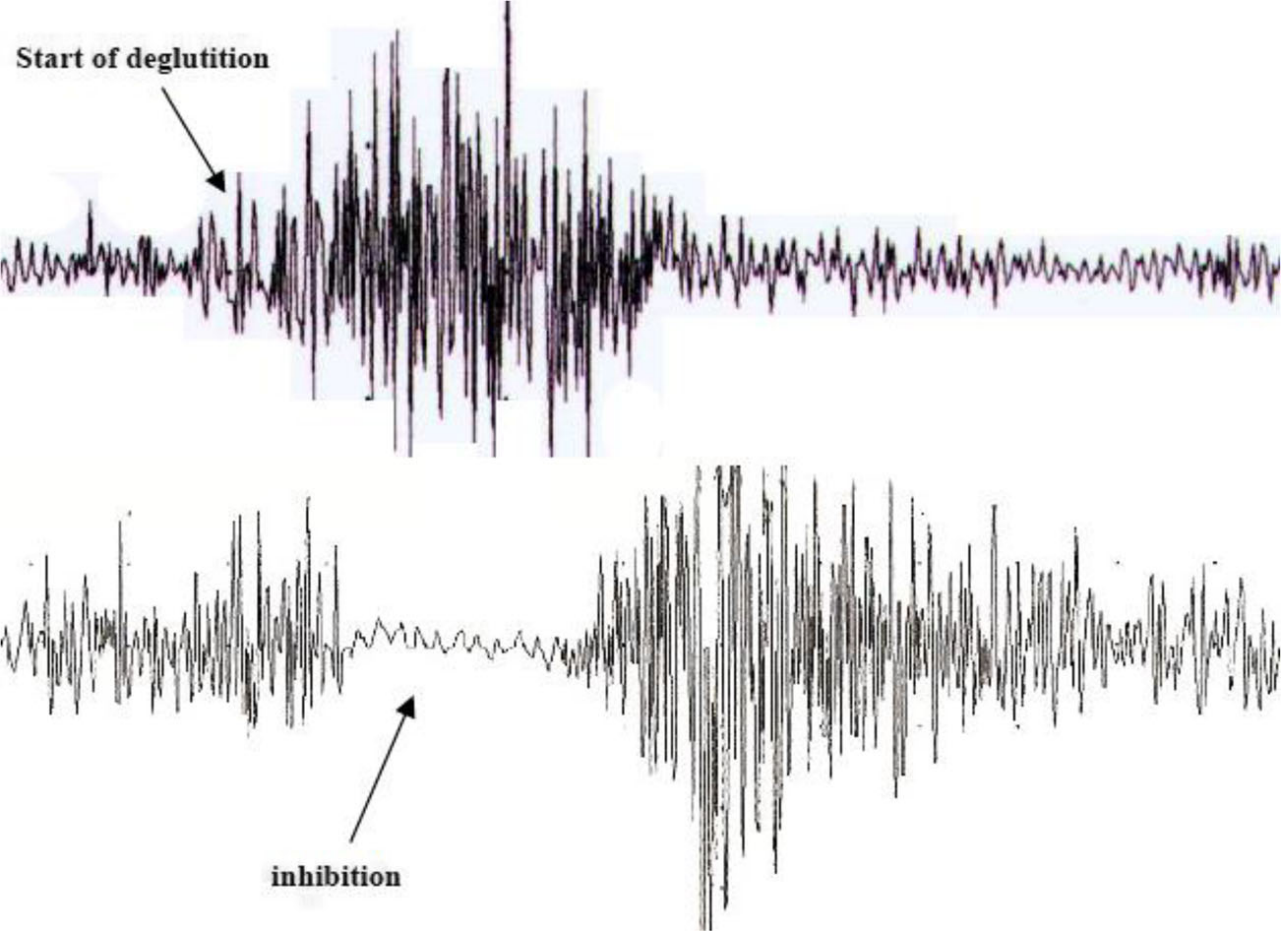
The figure shows the central program generator (CPG) located in the medulla oblongata and corresponding to the nucleus tractus solitaries (NTS), which receive both ascending and descending inputs and project to the ipsilateral brainstem nucleus such as nucleus ambiguous (NA); nucleus of V, VII, IX, X and XII cranial nerves; and C1–C3 tract of the cervical medulla. The existence of two CPGs can explain the recovery of the swallowing function after lesion of one CPG

In the forebrain, the most important areas deputed to swallowing are the anterior insula cortex and the frontoparietal operculum, including the inferior part of the sensorimotor cortex and a part of the premotor cortex. The corticobulbar connections originate in these areas and project to the ipsilateral and contralateral brainstem nuclei of the main cranial nerves involved in swallowing. The “central pattern generator” (CPG) for swallowing is located in the medulla oblongata, corresponding to the area of the nucleus tractus solitarius (NTS) [19]. CPG consists of four units, two per side, which receive both ascending and descending inputs and lead the final stage of the swallow. The NTS receives afferences from the nucleus ambiguous (NA), which in turn sends efferent fibers to the most important muscles for swallowing. Moreover, NTS receives sensitive inputs from oral, pharyngeal, and laryngeal mucosa as well as from upper cerebral areas and can modulate swallowing dependent on bolus properties such as size, texture, and temperature. A lesion which interrupts these connections thereby produces dysphagia. Furthermore, the two hemi-CPGs are tightly synchronized and organize the coordinate contraction of the bilateral muscles of the oropharyngeal region. Anatomical connections mediated by fibers crossing the midline have been found to exist between the two CPGs (see Fig. 1).

In ND, disturbances of the oral and/or pharyngeal phase are very frequent, in contrast to rarely occurring esophageal problems (oropharyngeal dysphagia). Dysphagia in patients with brainstem strokes such as Wallenberg syndrome or multiple sclerosis is usually caused by a focal lesion which interrupts the connections between the NTS and the NA ipsilateral to the lesion. Within 7–10 days, partial or complete recover occurs spontaneously due to vicarious function of the contralateral CPG. Instead, the dysphagia in patients with Parkinson’s disease and parkinsonisms involves pedunculopontine tegmental nucleus (PPTN) and dorsal motor nucleus of the vagus (DMV). More rarely, a CPG dysfunction with consequent increase of the inhibitory output to PPTN with lack of coordination of swallowing muscles can occur in PD. Within the esophageal myenteric plexus, however, Lewy bodies have been identified.

Peripherally, the swallowing is subdivided into 3 phases: oral phase, pharyngeal phase, and esophageal phase. The oral phase is accepted as voluntary, the pharyngeal phase is considered a reflex response, and the esophageal phase is mainly under dual control of the somatic and autonomic nervous systems [20]. The *oral phase s*tarts when the food is manipulated in the month and masticated; its primary function is the movement of the tongue and then the contraction of the lips and cheek muscles, orbicularis oris muscles, and buccinator muscles. The *pharyngeal phase* of deglutition involves not only pharyngeal and laryngeal muscles but also the muscles in the oral cavity such as tongue and suprahyoid muscles. The periorbital muscles actively contribute to the involuntary swallows. The actual motor events of swallowing can best be described as being composed of two phases: an oropharyngeal phase and a subsequent esophageal phase. The duration of the whole oropharyngeal sequence of swallowing lasts about 0.6–1.0 s The *esophageal phase* of swallowing is slower, may exceed 10 s, and consists of a peristaltic wave of contraction of the striated and smooth muscles, which propagates to the stomach.

### Diagnosis

Assessment of ND includes medical history, physical exam, and instrumental examinations.

### Clinical swallow evaluation

(1) Note the quality and sound of the patient’s voice: “Wet voice” may suggest long-term laryngeal aspiration, while a weak, breathy voice may indicate vocal cord pathology. (2) Inspection of soft palate and mouth, tongue and lips using a tongue blade, and handheld mirror allow to detect abnormality in motor function. (3) Normal laryngeal ascent can be palpated by placing the index finger above the patient’s thyroid cartilage when the patient swallows. The cartilage should move cephalad against the physician’s finger. (4) Observation of movements of the patient’s jaw, thereby patient mastication and patient capacity to mix food and saliva and push the bolus toward posterior pharynx, without choking or coughing. (5) Note an excess of saliva in the mouth with consequent stagnation. Sialorrhea or hypersalivation is not always due to excessive production of saliva. In fact, hypersalivation and drooling are often associated with impairment of swallowing coordination, and they are known to be associated with several neurological disorders (in Parkinson’s disease, its frequency varies from 10 to 84%) [21]. (6) Ask the patient to swallow and note any difficulty doing so. Observing the patient swallowing a variety of liquids and solids can be helpful to understand what type of dysphagia it is. Theoretically, dysphagia for solids suggests mechanical obstruction caused by diseases involving the esophagus or the base of the tongue (e.g., cancer or lymphoma). Dysphagia for liquids suggests a neurogenic dysphagia. Usually, dysphagia associated with degenerative diseases starts as dysphagia for liquids, but, over time, it becomes dysphagia mixed for liquids and solids.

Clinical examination to assess the dysphagia including “bedside swallowing examination” (BsSE) can be performed at the bedside by nurses. During “water swallowing test,” the patient is asked to swallow 50 ml of water in 5-ml aliquots. ND is diagnosed if the patient chokes or coughs or if any alteration in the voice quality is detected. If the patient drinks all 50 ml of water without symptoms, he is considered to swallow normally. Patients with silent aspiration may have no problems during this test. Several assessment tools [22] are used to evaluate and quantify the dysphagia: (1) Swallowing Disturbance Questionnaire, a self-reported 15-item questionnaire on swallowing disturbances; (2) Eating Assessment Tool (EAT-10), a self-administered, symptom-specific outcome instrument for dysphagia; (3) Dysphagia Outcome and Severity Scale (DOSS), a 7-point scale developed to systematically rate the functional severity of dysphagia based on objective assessment; and (4) Penetration-Aspiration Scale (PAS), an 8-point scale based on VFS, to describe penetration and aspiration events. This is the most used scale for semi-quantitatively assessing the degree of endoscopically and radiologically measured penetration/aspiration.

### Instrumental assessment

#### Fiberoptic endoscopic evaluation of swallowing (FEES)

FEES is an assessment using a flexible nasendoscopy, which is passed into the nares, over the velum and into the pharynx, in order to perform a functional and morphological study of the velopharyngeal sphincter and assess pharyngeal and laryngeal reflexes. Various aspects of swallowing are observed directly using colored water and a solid bolus. FEES permits the detection of abnormalities of swallowing, laryngeal penetration of bolus, or tracheal aspiration. This procedure permits the evaluation of the efficiency of changes in posture during rehabilitation in patient with ND [23].

#### Videofluoroscopic swallowing study (VFSS)

VFSS is a useful tool for determining the presence, severity, and characteristics of dysphagia [24]. The patient ingests bolus mixed with radiopaque substance, such as barium, using a range of food and fluid consistencies. The VFS is viewed on a monitor/screen and recorded allowing a dynamic exploration of swallowing process from the bolus formation in the oral cavity to the entrance through the esophageal sphincter to the stomach. Videofluoroscopy with modified barium swallow (MBS) is a more commonly used instrumental assessment for evaluation of swallowing. Studies using MBS testing have demonstrated subclinical alterations in the oropharyngeal phase of swallowing in 75–97% of patients with PD. Pharyngeal manometry complements the MBS with videofluoroscopy in diagnosing pressure-related causes of dysphagia. Despite its utility in confirming disorders of the upper esophageal sphincter (UES) or pharyngeal constriction, the use of pharyngeal manometry is limited for research purpose due to its complexity. It was postulated that FEES and VFSS have a sensibility of 80–90% and a specificity of 50% for detecting aspiration in patients with stroke and other ND.

#### Electromyography (EMG)

**Fig. 2:**
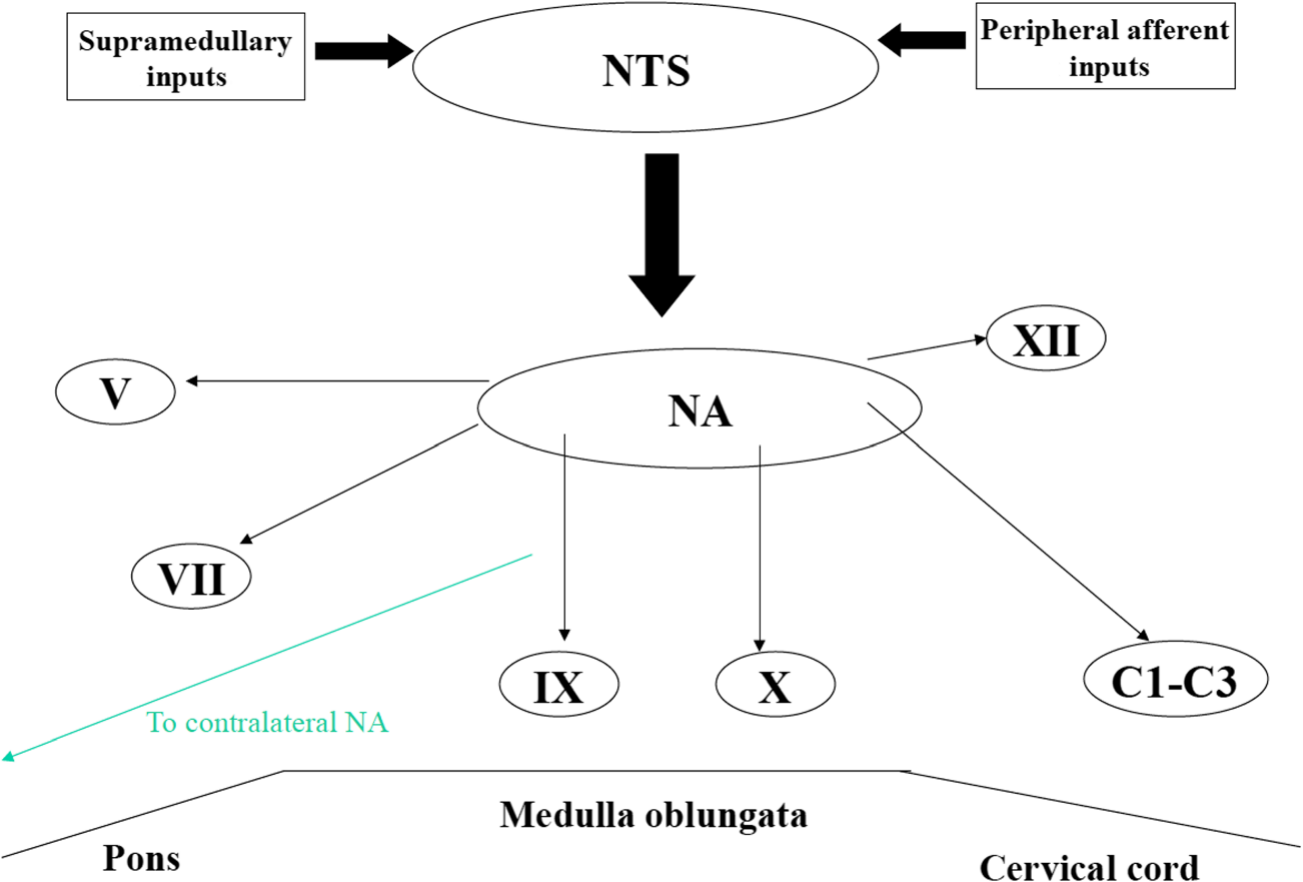
The figure shows EMG recording of the IC (top trace) and CP (bottom trace) in a health subject during voluntary swallowing, using concentric needle electronic. At the activation of the IC corresponds a relaxation of UES allowing that the bolus passes from the pharynx to the esophagus. Calibration: gain, 200 μ V/D; sweep, 500 ms/D

EMG study is very useful in clinical diagnosis to understand the physiology of activation of muscles involved in oropharyngeal phase of swallowing and also to identify target deglutition muscles for infiltrating with botulinum toxin [25]. There are several groups of muscles of deglutition that can be studied in detail: (1) jaw and perioral muscles; (2) submandibular/suprahyoid (SM) muscles; (3) tongue muscles; (4) laryngeal and pharyngeal muscles; and (5) cricopharyngeal (CP) muscle of the upper esophageal sphincter (UES). Ertekin and al. [26] have established a technique for recording the activity of SM muscles by superficial electrodes and the thyroarytenoid muscle and CP muscle by concentric needle electrode, while the subject swallows 2–3 ml of water. Furthermore, a piezoelectric accelerometer (transducer) connected to an electromyograph is localized to the thyroid cartilage. This accelerometer follows hyolaryngeal excursion movements. Surface EMG activity of the SM muscles gives a considerable amount of information about the onset and duration of the oropharyngeal swallowing, because the contraction of the SM muscles pulls up the hyoid bone into an anterosuperior position, which elevates the larynx and initiates other reflexive changes that constitute the pharyngeal phase of swallowing. When a swallow is initiated voluntarily, the contraction of the SM muscles should be controlled by at least two routes. During the initial part, SM muscles should be activated by the cortical drive either directly or via the brain stem CPG. The latter part of SM muscle activation should, however, be controlled by the CPG of the brain stem network, especially in the period immediately after the onset of laryngeal upward movement, which is an important and early event of the pharyngeal phase in voluntarily induced deglutition. When the larynx is pulled up anterosuperiorly by the SM muscles during the pharyngeal phase of swallowing, the laryngeal adductor muscles are activated for the closure of the vocal cords. By this mechanism, the larynx and lower airways are thought to be protected from swallowing bolus that is passing through the pharynx. Thus, the activities of both groups of muscles are interrelated through the CPG of the swallowing program. Laryngeal adductor muscles including the thyroarytenoid muscle are mainly activated for the protection of the larynx during swallowing. The protective activity of the laryngeal adductors usually begins after the contraction of the SM muscles in both voluntarily initiated and spontaneous reflex swallows. The CP sphincter muscle is tonically active during rest, and this continuous activity ceases during a swallow in human subjects. Usually, it is preferred simultaneous recording of IC and CP muscle of the UES using concentric needle electronic (Fig. 2) and also of SM and perioral muscles, when it is necessary. The choice of recording from IC is due to the fact that this is the last pharyngeal muscle temporarily involved in the propulsion of the bolus toward the esophagus, because it is located in direct contact with CP. At the activation of IC corresponds a relaxation of UES. This period 0,6 msec, the bolus passes from pharynx to esophagus via UES. IC control is totally voluntary and represents the input of the reflex relaxation of UES [27].

### Management of dysphagia

The main swallowing disturbances are secondary to reduced lingual control, alterations of movements mostly impaired tongue base retraction, delayed or absent pharyngeal swallow reflex, reduced pharyngeal contraction associated to bradykinesia, cricopharyngeal dysfunction, and reduced laryngeal closure (due to a decreased SM muscles activity). These disorders may be found alone or in association with each other.

### Rehabilitation therapy

A rehabilitation treatment plan is decided after evaluation of patient’s cognitive, motor, and sensory abilities and the impact of these impaired abilities on the recovery of swallowing function. Generally, these are patients with stroke, because it is the most extensively studied neurological condition.

Behavioral intervention such as voluntary airway closure techniques or other types of swallowing training may be useful in individual cases [28], even if the use of specific maneuvers may request help to the caregivers. Anyway, there are no controlled clinical studies.

Functional therapy is divided into restitution, compensation, and adaptation methods [29]. (I) Restitution focuses on partial or complete restitution of disturbed functions. Effortful swallowing exercise is indicated for patients with an impaired tongue base retraction and/or reduced pharyngeal propulsion. Stimulating the anterior faucial pillars effectively triggers the swallowing reflex. The combination of mechanical, thermal, and gustatory stimuli seems to be more efficient. (II) Compensation includes postural changes and swallowing maneuvers. The patient should sit on a chair in a comfortable way, usually upright position, while eating and drinking. In patients who have difficulty triggering the swallowing reflex, tilting the head forward during eating may avoid leaking of the bolus and subsequent aspiration. When tongue movements are impaired, resulting in difficulty initiating a swallow, but the pharyngeal phase of swallowing is intact, tilting the head backwards helps guide the bolus into the pharynx. The Mendelsohn maneuver is a technique that helps open the UES and prolong its opening time. The patient has to hold the upward movement of the larynx during swallowing for some seconds. This maneuver is appropriate for patients with pharyngeal residues or deficient opening of the UES. (III) Adaptation means modifying the type of food to ease nutrition. Dietary modification may help prevent extremely long mealtimes, fatigue, and dread of meals. Soft textures or puréed food can compensate for a poor oral preparation phase and ease oral and pharyngeal transport. Liquids should be thickened, e.g., with gel water, if thin drinks cause choking. Triggering the swallowing reflex can be enhanced by emphasizing taste or temperature; cooled drinks are often easier to swallow.

### Pharmacological and non-pharmacological therapies

Pharmacological treatment of these problems includes oral anticholinergic drugs [30].

Surgical myotomy of the CP, primary muscle of the UES, showed an important improvement of oropharyngeal dysphagia associated to UES hyperactivity and should be considered treatment of choice in selected cases with cricopharyngeal dysfunction [31]. Surgical therapy should have a limited use because it is performed under general anesthesia and is unsafe in debilitated patients.

In the last few years, chemical myotomy of UES by local injections of botulinum toxin type A (BTX-A) into the cricopharyngeal (CP) muscle of the UES has been proposed as an alternative, less invasive, and less unsafe than surgical myotomy. This technique showed to be efficient and without significant side effects for the treatment of oropharyngeal dysphagia associated with different neurological and no neurological diseases, characterized by hyperactivity or failure/reduced relaxation of the UES. The advantages of this treatment are safety and repeatability, and it can be still performed in debilitated patients, because general anesthesia is not necessary. Furthermore, it can be used as a test of efficiency for a possible following surgical myotomy that may be effective in about 25% of patients. In fact, it has been established that patients who respond positively to the chemical myotomy respond positively to the surgical myotomy [24, 32–34]. The disadvantages derive from the fact that this technique needs to be performed by an expert operator and that toxin can spread to neighboring laryngeal muscles.

As the dysphagia becomes more severe, introduction of nasogastric tube (NGT) or a percutaneous endoscopic gastrostomy (PEG) should be considered. NGT is only indicated in patients who have the inability to eat or drink enough, thereby in persons who are threatened by malnutrition and dehydration. NGT is indicated for patients with acute diseases such as stroke or head injury in which the dysphagia may disappear within weeks or months. In fact NGT can cause frequent side effects (erosion of mucous, kinking, and shifts of the tube). PEG is more suitable for patients with degenerative diseases [35] such as Parkinson’s disease (PD), parkinsonisms, dementia, or amyotrophic lateral sclerosis (ALS) in which the goal is to feed the patient for a long time (months or years). The complications with this technique vary from minor complications (about 20%) such as local pain or skin infections to major complications such as peritonitis and pneumonia (about 1–3%).
